# A Welfare Assessment Tool to Harmonize Care and Management for Research Rabbits

**DOI:** 10.3390/ani16081229

**Published:** 2026-04-17

**Authors:** Carly I. O’Malley, Sarah E. Thurston, Elizabeth A. Nunamaker

**Affiliations:** Charles River, 251 Ballardvale St., Wilmington, MA 01887, USA; carly.omalley@crl.com (C.I.O.); sarah.thurston@crl.com (S.E.T.)

**Keywords:** rabbits, welfare, research, welfare assessment tool

## Abstract

Animal welfare assessment is a vital component of working with animals in a research environment and is a growing expectation. Rabbits are a common species worked with in biomedical research, but information on rabbit welfare and best practices are limited, making it difficult to assess welfare. To address this gap, a rabbit welfare assessment tool was developed within an international contract research organization and commercial breeder. The tool contained 134 descriptors including input- and output-based measures of welfare across six categories: physical, behavioral, environmental, training, procedural, and culture of care. Benchmarking assessments occurred in March and September 2025, assessing 13 facilities across seven countries. In March, overall scores were in the range of 70–92%, with high scores in the physical category (avg: 97%) and the most room for improvement in training (avg: 73%). In September, overall scores were in the range of 77–88%, with the highest score in the physical category (avg: 98%) and the most room for improvement in culture of care (avg: 74%). The results of the assessments identified strengths in rabbit management programs, as well as identified areas needing refinement. The results allow for development of facility and organizational level goals to improve rabbit welfare and create a culture of continuous improvement.

## Simple Summary

Animal welfare assessment is an important part of managing research animals. Animal welfare assessment tools help to standardize welfare criteria to ensure consistent, holistic assessments. Rabbits are an important biomedical model but information on optimal practices for rabbit welfare within a research environment are limited. To meet this gap, a rabbit welfare assessment tool was developed as part of a Rabbit Welfare Working Group within an international contract research organization. The tool contained 134 welfare descriptors across six categories: physical, behavioral, environmental, training, procedural, and culture of care. The tool was launched within the organization, assessing 13 facilities across seven countries. Benchmarking assessments occurred in March and September 2025. The tool aided facilities in identifying strengths and weaknesses in their rabbit management programs, allowing for targeting goal setting for program improvement and creating a culture of continuous improvement for animal welfare.

## 1. Introduction

Rabbits are a common species worked with in biomedical research, especially in pharmacological [1], cardiovascular [2], vaccine development [3], ocular [4], and embryo-fetal development [5] studies. Within the U.S., rabbits were the third most worked with species in 2024 [6], and their use continues to grow within the EU as well [7]. Rabbits are often used as a second, non-rodent species for studies requiring regulatory testing [8]. As work with dogs and primates becomes more restricted [9], work with rabbits will likely continue to grow. While working with rabbits in a research environment can present benefits in terms of housing, size, and breeding ease, rabbits present challenges with handling and restraint [10,11,12,13,14] and social housing [15]. Additionally, despite their regular use in research environments, information on optimal practices for improving rabbit welfare is scarce.

When working with research animals, monitoring and assessing animal welfare should be standard practice, and there are growing expectations from study sponsors and regulatory requirements for animal welfare assessment [16]. Local animal ethics committees, third-party animal welfare assessors, or national ethical guidelines may outline criteria to consider for animal welfare assessment [16,17,18,19], but interpretation and implementation is largely left to the institution. There are different models of welfare that could be considered, such as the three components model of welfare [20], Five Freedoms model [21], and Five Domains model [22]. Each model may offer similar, but varying suggestions on what factors should be considered important to animal welfare. Which of these are considered and prioritized may differ based on experience, education, research purpose, culture, and region [23,24].

Implementing welfare assessment tools can help to harmonize welfare assessment across research groups and facilities by creating common criteria and language around welfare. Welfare assessment tools can also help provide a quantitative measure of welfare that can be used to identify needed refinements and monitor the impacts of programmatic changes over time. Welfare assessment tools should aim to be holistic and comprehensive, considering both input- and output-based measures of welfare [25,26]. Input-based measures are what we provide to the animals, such as housing, resources, and social companions [25,27]. Output-based measures are measures taken directly from the animal, such as body condition, animal behavior, and hormonal measures of stress [26].

There are some welfare assessment tools developed and available, such as Welfare Quality^®^, but these tend to be geared toward livestock species such as pigs [28], cattle [29], and laying hens [30]. The Welfare Quality protocol was adapted for use in meat rabbits [31,32]. Additionally, welfare impact scoring criteria were developed and compared for meat rabbit across housing systems [33], and a welfare assessment protocol was developed for pet rabbits [34], but there are no welfare assessment criteria or tools specific to research rabbits. Previously, a welfare assessment tool for research primates was developed [35]. Based on the positive outcomes and feedback from that tool, additional species tools were developed, including rabbits. The goal of this project was to develop a rabbit welfare assessment tool that could be implemented across a global contract research organization that does commercial breeding, discovery research, and safety testing with rabbits. The aims of this paper are to describe the development of the rabbit welfare assessment tool, provide an overview of the tool, and discuss the results and outcomes of the assessment.

## 2. Materials and Methods

All sites housing rabbits within the organization were AAALAC-accredited and met or exceeded country- or region-specific requirements for animal care and welfare. Animals assessed using the welfare assessment tool were covered under site- and/or protocol-specific animal care and use protocols.

### 2.1. Tool Development

The development of the rabbit welfare assessment tool followed previous work with a primate welfare assessment tool, described in Paterson et al., 2024 [35]. The rabbit welfare assessment tool was developed and implemented by Charles River, led by Global Animal Welfare, which is a corporate animal welfare oversight group. To improve rabbit welfare and harmonize optimal practices when working with rabbits, a Rabbit Welfare Working Group was formed. The group was formed in June 2023 and completed discussions in March 2024. The full details of the working group is described by Thurston and colleagues [36]. Briefly, the Rabbit Welfare Working Group consisted of rabbit experts split into subcommittees based on topic area, including one specific to rabbit welfare assessment [36].

The framework of the tool was based on the primate welfare assessment tool [35], with the welfare descriptors and welfare weights being adapted to reflect the welfare needs of rabbits based on the available literature and expert opinion from the Rabbit Welfare Working Group where there were gaps in the literature. The main researchers (CIO, SET) developed descriptors based on rabbit welfare criteria. The draft tool was then presented to members of the subcommittee on Animal Welfare Assessment for review and feedback. The subcommittee included 13 members across 9 sites and 6 countries (US, Canada, France, Netherlands, Ireland, and Scotland). The subcommittee members were all subject matter experts in rabbit welfare and included an animal behavior and welfare scientist, behavior and welfare technicians and program coordinators, technical trainers, and a clinical veterinarian. The subcommittee members reviewed the draft tool and provided feedback on the descriptors and welfare weights. The preliminary tool was built in Microsoft Excel (Microsoft Corporation, Redmond, WA, USA, Microsoft 365 version).

The descriptors were the individual items being scored. The welfare weights ranged from 1 to 5 based on importance to rabbit welfare, with items ascribed a welfare weight of 5 being items that are most important to welfare and 1 being items less important for rabbit welfare. The scores were numeric, including 0, 1, or 2. A score of 0 indicated never or disagree, 1 is sometimes or somewhat agree, and 2 is always or agree. A few descriptors had binary scores of “yes” or “no”, and some items had “NA” or “non-applicable” as an option. Non-applicable was used in situations where a descriptor was not possible at that site or could not be observed during the time of assessment, such as observing social behaviors or social compatibility if the animals were singly housed (facilities would have to put a score of 0 for “Animals are housed with at least one other compatible social partner” but could not otherwise assess social behavior if animals are singly housed), or parental behavior if breeding did not occur at the site. There were bonus items as well for items that were beneficial to rabbit welfare but were not possible at most sites, such as having remote video monitoring to observe animals or providing rabbits substrate to dig in. The forms of the tool, including the scoring criteria for each descriptor, are included in Supplementary Materials.

An overview of the welfare categories, subcategories, and maximum scores per welfare subcategory is outlined in Table 1, with the full tool provided in Table 2. The assessment was completed across three forms, including a room-level form that includes descriptors scored within animal rooms using animal-based and resource-based indicators while observing the animals in their home environment; the site-level form that assesses over facility protocols; and a culture of care form that is a survey provided to employees working with rabbits to assess employee training, resources, and well-being.

### 2.2. Tool Launch

The final version of the tool was translated into French, Hungarian, and Dutch. The translations were conducted in DeepL (DeepL SE, Cologne, Germany), and then reviewed by people fluent in each language to ensure accuracy. The tool was then created in Smartsheet (Smartsheet Inc., Bellevue, WA, USA, version 2024) to allow for the welfare assessment data to be submitted into Smartsheet. Forms were created for room, site, and culture of care and in each language. The data entered with the forms was collected into a worksheet, which took the score entered and multiplied by the welfare weight for each descriptor. The data collected in Smartsheet had a live connection with PowerBI (Microsoft Corporation, Redmond, WA, USA, version 2024), where there were equations to summarize the data for each subcategory and category per site, and display the results in visual dashboards. The dashboards allowed tool users to filter data based on category, subcategory, time frame, year, and facility, as well as to review data inputs and sort and filter based on tool descriptors or scores. This method allowed for automated data analysis and visualization, providing results to tool users as soon as they had finished their assessment.

Facilities with rabbits were asked to identify 1–3 tool users. Tool users were assigned to tool-use training, which was provided in the previously listed languages. The training was presented as an e-learning module that took 10–15 min to complete. The training was generic to species to allow training to be used across all species tools. The training includes learning outcomes, purpose of the welfare assessment tools, what is included in the tools, how to complete the assessment, and a knowledge check. The knowledge check is five questions. Tool users needed to achieve 100% on the knowledge check to successfully complete training. Tool users who had completed the training were provided access to the Smartsheet forms and a Teams (Microsoft Corporation, Redmond, WA, USA) channel that contained links to the Smartsheet forms, printable versions of the forms, and instructions on how to access the PowerBI report dashboard.

Tool users were instructed to complete one site-level form, room-level assessments for approximately 10% of their rabbit rooms (minimum 3 rooms, or total number of rooms if less than 3, and a maximum of 10 rooms), and 3–6 culture of care assessments by sending the link to the form to employees who work with rabbits. For the room-level forms, tool users were instructed to have the assessments reflect the rabbit population at the facility, considering breeds, study types, short- and long-term studies, non-GLP and GLP studies, and include colony or training stock, where relevant. Similarly, tool users were instructed to recruit short- and long-term employees who work with rabbits in different roles (i.e., husbandry personnel, study technician, veterinarian) for the culture of care surveys. The surveys were completely anonymous to understand opportunities for local improvement only. As such, ethical review was not required under institutional policy.

The rabbit welfare assessment tool was launched company-wide in March 2025 (Q1) with a second assessment conducted in September 2025 (Q3). These assessments aimed to collect baseline data to allow sites to understand how to use the tool and provide a benchmark for future assessments, to allow sites to monitor their progress over time. The same tool users completed both assessments.

EndNote 21 (Clarivate, Philadelphia, PA, USA) was used to manage the reference library.

## 3. Results

**Figure 1:**
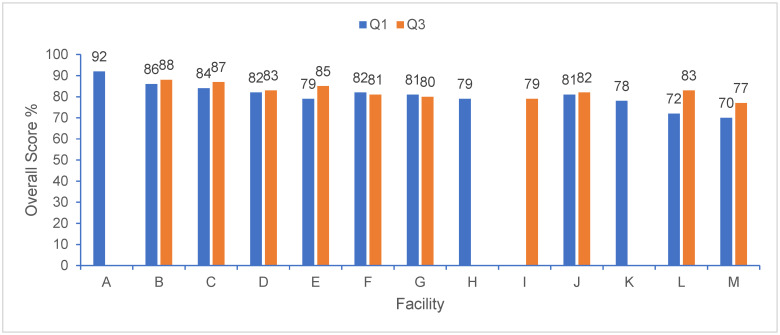
Overall rabbit welfare assessment scores (presented as percentage based on score achieved/total maximum score) for the first two assessments in March 2025 (Q1) and September 2025 (Q3).

An overview of the overall scores for each facility for each assessment is summarized in Figure 1. At the time of the benchmarking assessments, there were 13 facilities in the company with rabbits. In March 2025 (Q1), 12 facilities participated in the rabbit welfare assessment, with overall scores being in the range of 70–92%. There were 16,238 animals assessed across 31 rooms, and a total of 39 employees completed the culture of care assessment. When looking at the global overall scores, the highest scoring category was physical (97%), followed by behavioral (87%), procedural (76%), culture of care (75%), environmental (75%), and training (73%). In September 2025 (Q3), 10 facilities participated with scores in the range of 77–88%, assessing 3927 animals across 23 rooms, with 36 employees assessed. Globally, the highest scoring category was again physical (98%), followed by behavioral (88%), procedural (81%), environmental (77%), training (76%), and culture of care (74%). The full results for each assessment for each facility are provided in Table 3. General findings for each category are discussed below.

**Physical.** The physical category assessed the clinical health of the rabbit and factors that may influence health and included the subcategories general condition, nutrition, pain assessment and mitigation, and records. Overall scores in the physical category were in the range of 93–100% in Q1 and of 95–100% in Q3. The subcategory with the highest average score was general condition (Q1: 99%; Q3: 100%), which assessed posture, hair coat, body condition, teeth, and whether animals appear well hydrated, are responding normally to external stimuli, and are free of obvious wounds or health concerns. The subcategory with the lowest average score was pain assessment and mitigation (Q1: 92%; Q3: 89%), where the descriptor “care personnel are trained in recognizing species-specific signs of pain” was scored with a “1” for three facilities, suggesting the need for more rabbit-specific training.

**Behavioral.** The behavioral category assessed normal and abnormal rabbit behavior, including behavioral assessments, normal behavior, and social, feeding, and parental behaviors. Overall scores were in the range of 76–96% in Q1 and of 72–97% in Q3. The subcategory normal behavior had the highest average scores (Q1: 99%; Q3: 99%). In Q1, the lowest score subcategory (Q1: 74%) was feeding behavior, which assessed whether food was provided in a way to encourage natural behaviors, but in Q3, this subcategory increased to 87%, suggesting facilities used the results to implement more natural feeding opportunities for rabbits. The other lower scoring subcategories were behavioral assessments (Q1: 81%; Q3: 72%) and social behavior (Q1: 80%; Q3: 82%). For behavioral assessments, the results indicate a need for regular behavioral assessments of rabbits. Four facilities indicated that behavioral assessments are not regularly conducted for rabbits, and five indicated sometimes or for some animals (score of “1”). For social behavior, the scores reflected that single housing rabbits is common practice due to study protocols and/or difficulty managing socially housed rabbits. Many of the social behavior descriptors were scored as “NA”. For those facilities that did socially house rabbits, the scores reflected no or inconsistent social behavior monitoring for compatibility. Only 6 of the 13 facilities scored above 80% for social behavior, indicating a crucial opportunity for improvement. One site received bonus points for having remote video monitoring for observing animals.

**Environmental.** The environmental category assessed the quality of the space and resources and the ability for rabbits to express species-typical behaviors and postures. It included housing, resources, and exercise opportunities. The overall scores were in the range of 48–98% in Q1 and of 55–95% in Q3. The subcategory with the highest average scores was resources (Q1: 82%; Q3: 84%), followed by housing (Q1: 76%; Q3: 77%), then exercise opportunities (Q1: 40%; Q3: 41%). In the resources subcategory, facilities most often struggled with having formal behavioral management programs for rabbits, where there were guidelines, rotating resource schedules, documentation, and evaluation. Providing thermoneutral surfaces in the rabbit enclosures was also an identified area for improvement. For the housing subcategory, providing visual barriers within the environment and substrate were the biggest challenges. Three sites received bonus points for providing substrate for the animals to dig in.

**Training.** The training category assessed how rabbits were prepared for the research environmental to reduce stress and promote cooperation, including acclimation, habituation and training, human interactions, and animal cooperation. The overall scores were in the range of 47–91% in Q1 and 52–94% in Q3. The subcategory with the highest average score was acclimation (Q1: 96%; Q3: 100%), with all facilities providing an acclimation period after arrival to the facility before study procedures started by the Q3 assessment. The lowest scoring subcategory was habituation and training (Q1: 60%; Q3: 65%) followed by human interactions (Q1: 73%; Q3: 76%), highlighting the need for more formal programs with rabbits to encourage voluntary cooperation and positive human–rabbit interactions.

**Procedural.** The procedural category assessed the procedures and techniques used at the facility to ensure they are in line with the recent literature on optimal practices for rabbits. The overall scores were in the range of 57–88% in Q1 and of 60–94% in Q3. The subcategories with the highest average scores were ambience (Q1: 85%; Q3: 88%) and restraint (Q1: 88%; Q3: 85%). Scheduling was the biggest challenge (Q1: 63%; Q3: 56%), indicating that personnel felt rushed. Only 6 of the 13 sites responded with scores of “2”, stating there were sufficient personnel scheduled to avoid feeling rushed. The procedures subcategory also identified needed refinements (Q1: 64%; Q3: 75%) in providing post-procedure rewards. Facilities additionally lost points in this subcategory due to lack of compatible social partners. Eight facilities received bonus points for preparing animals for adoption prior to rehoming.

**Culture of care.** The culture of care category assessed the training and resources provided to employees working with rabbits. This included initial training, continuing education, compassion fatigue and resiliency building programs and activities, involvement and opportunity, choice and control in work schedule, recognition, and competencies. The overall scores were in the range of 66–97% in Q1 and of 62–95% in Q3. In Q1, there were 39 employees assessed. A total of 20 identified as women, 7 identified as men, and 12 chose not to answer. For length of time working at the site, 5 worked less than 1 year, 12 worked for 1–5 years, 11 worked from 6–10 years, and 11 worked for over 10 years. In Q3, there were 36 employees assessed. Of the employees assessed, 20 were women, 14 were men, and 2 preferred not to respond. A total of 4 of the employees worked at the site for less than a year, 15 worked 1–5 years, 7 worked 6–10 years, and 10 worked over 10 years. The subcategory with the highest average scores was initial training (Q1: 87%; Q3: 84%). The lowest scoring subcategories were choice and control (Q1: 65%; Q3: 57%) and compassion fatigue and resiliency building programs/ activities (CF/RBA) (Q1: 63%, Q3: 67%). For choice and control, the descriptors relate to whether personnel can choose to specialize with a preferred species, whether they can opt out of performing activities they are uncomfortable with, and if they feel rushed. There were mixed responses for each item across facilities (responses of 0, 1, and 2). For the CF/RBA subcategory, there were again mixed responses of 0, 1, and 2 across the descriptors that include whether personnel could identify their Resiliency Building Advocates, had good work–life balance, could step away when feeling overwhelmed, had resources on compassion fatigue and resiliency building, facility had an active CF/RBA program, and the facility had opportunities to honor the animals they work with.

**Recommendations.** Based on the results of the benchmarking rabbit welfare assessments, recommendations were developed for how to address gaps in rabbit management programs. These recommendations are provided in Table 4.

## 4. Discussion

The goal of this project was to develop a rabbit welfare assessment tool for animals in varied research environments, including commercial breeding, discovery research, and safety testing. A rabbit welfare assessment tool was successfully implemented across 13 facilities in a global contract research organization and commercial breeder. The tool included input- and outcome-based measures of animal welfare and personnel wellbeing to ensure holistic assessment of factors that influence research rabbit welfare. The tool helped to identify facility- and company-level strengths and weaknesses in rabbit management and resulted in global recommendations, goal setting, and action items to address gaps and create opportunities for improvement.

The results of the assessment highlight key areas where rabbit management programs could be refined. More details on key recommendations for improving rabbit welfare in a research environment are provided by Thurston and colleagues [36]. The main challenge found in developing criteria and recommendations for improved rabbit welfare is a lack of research for rabbits in this environment. At the time of this publication, much of the published literature on rabbit welfare is on meat rabbits [25,31,32,33,37,38] or pet rabbits [34,39,40]. There are few empirical studies investigating refinements for research rabbits. As their use in biomedical studies continues to be important, more research is needed to better understand how management practices impact rabbit welfare.

Welfare assessment criteria has been developed for use in meat rabbits [31,32] and pet rabbits [34], but due to differences in how animals are housed and worked with in a research setting, previously developed tools did not meet the needs of the company. For meat rabbits, the Welfare Quality protocol was adapted for rabbits where the criteria focused on absence of negative experiences (i.e., absence of injuries; absence of prolonged hunger) and included environmental criteria such as cleanliness and height of drinkers, light quality, and temperature [31,32]. All the facilities assessed with the tool described in this paper were AAALAC-accredited; therefore, they already meet or exceed a minimum standard in terms of housing and environment. Additionally, the goal was to move beyond assessing the absence of negative behaviors and factoring in positive experiences [41] and comprehensive behavioral management [42]. For the behavioral parameters under the Welfare Quality criteria for rabbits, biting and isolation were the two criteria for social behavior, and human–animal relationship included a human approach test, training, and touching kits [31,32]. In contrast, the current described tool had more emphasis on observing normal behavior and positive social behaviors, regularly assessing behavior and compatibility of socially housed animals for proactive animal management, and the training category focused on building cooperation with the animals through habituation and positive reinforcement training. Research animals are used for a variety of purposes and study procedures that involve regular human–animal interactions, which is why assessing animal training, procedural refinements, and culture of care (looking at personnel training and resources) were important additions for welfare assessment of research animals. Additionally, due to previous welfare assessment tools within the organization, the same framework was implemented for rabbits to aid in communication, training, and ease of use for personnel using the tools. While the framework of the tool is the same, each tool is fully adapted for that particular species and reviewed by species experts.

The development of the rabbit welfare assessment tool came after previous efforts and feedback from implementation of a primate welfare assessment tool [35] and a dog welfare assessment tool. Each iteration of species-specific welfare assessment tools helped to refine the tools and messaging to ensure uptake and proper use of the assessment results in evaluating programs. Facilities were coached in reviewing and sharing results with key personnel at their facility (i.e., veterinarians, behavior groups, ethics committee members, operations personnel, site management) and identifying 1–3 goals for the following year. Feedback from the primate welfare assessment tool stated that the goal setting was helpful for sites to prioritize efforts and resources and improving communication towards common goals. The corporate animal welfare group followed up on goals annually through virtual check-ins and site visits. From a company level, the assessment results identified global gaps that resulted in working groups addressing those gaps (e.g., habituation harmonization, species housing improvement) and targeted training opportunities (e.g., animal welfare and 3Rs certificate program, behavioral management certificate program, species-specific workshops, discussions, on-demand virtual trainings).

The rabbit welfare assessment tool was the first tool to be used in commercial rabbit breeding facilities. Previous species tools were designed for animals used in discovery and safety testing research, which have different goals and priorities compared to the commercial breeding facilities. Additional considerations were needed to ensure the tool was effective at evaluating facilities from the three different business units. More descriptors had the opportunity to score “non-applicable” to accommodate the breeding facilities. Following the benchmarking assessments, personnel from the breeding facilities were asked to provide feedback and suggestions on the tool. Ultimately, they believed the tool was useful in evaluating their programs but indicated that additional descriptors needed to have the “non-applicable” option (personal communications). The non-applicable option was mainly provided for descriptors in the training and procedural categories, as the breeding facilities are not training animals for cooperation nor conducting procedures, (i.e., oral gavage, dermal applications, blood collection).

There are limitations of the tool. While the tool was designed to have input- and outcome-based measures of animal welfare, the tool cannot provide individual animal assessment, provide assessment on severity or cumulative use [43], or directly measure the effectiveness or quality of the programs on animal-based measures of welfare (i.e., is the behavioral assessment program effective at identifying and mitigating abnormal behaviors), but rather use the animal-based measured (i.e., abnormal behavior observed, evidence of fighting, animals show fear towards humans) to highlight programmatic or systemic animal management gaps that allow for targeted discussions, training, and resource allocation. Therefore, the tool may not directly improve an individual animal’s welfare at a given time point, but rather helps facilities and the organization identify issues and implement changes that will impact animals in the future (i.e., capital investment in updating animal housing, providing more time for habituation and training of animals prior to study start). It is also important to note that each facility has its own animal use protocol review process; therefore, the animals assessed with this tool would be additionally monitored based on the protocol they are under.

Additionally, using the tool can be time consuming, with tool users reporting that it can take 2–8 h depending on the size of the facility. With multiple species welfare assessment tools now being used within the company, the time commitment needed to complete the assessments can add up for facilities that have multiple species. Therefore, frequent assessment is not practical using this tool. The tool is best at evaluating systemic welfare risks in species-specific management programs and monitoring the impacts of programmatic changes over time. Initial assessments are conducted six months apart. Following the benchmarking assessments, facilities have the option to conduct the assessment one or two times during the calendar year. To truly have a holistic assessment of animal welfare, multiple welfare assessment tools may be needed to capture different factors that impact welfare at an individual animal and programmatic level. For example, in the CCAC guidelines for animal welfare assessment, it is stated that assessments should consider the whole life of the animals, monitor species normal and abnormal behaviors, identify systemic welfare risks and individual animal scientific, humane intervention, and cumulative endpoints, considering physical condition, psychological well-being, and experimental impacts [16]. To meet these criteria and have a comprehensive welfare assessment protocol that can allow for decision making at an individual and programmatic level, multiple types of welfare assessment tools need to be used. There also needs to be strong data collection, visualization, and communication pathways to ensure key personnel are involved and appropriate actions are taken to reduce risk and improve animal welfare [16,43].

Another current limitation of the tool is that formal measures of reliability and validity have not yet been conducted. Because the tools are developed by species experts based on empirical evidence and expert experience (where the literature is lacking), the tool has content validity. However, the tools were developed and released internally within an organization; therefore, obtaining external feedback would be necessary to confirm content validity. No formal measures of construct validity were assessed. Anecdotally, the rabbit welfare assessment scores across facilities typically reflect internal observations of animal management programs and what is observed on site visits, but no formal measures have been conducted. Because the tool has three separate forms measuring different aspects of welfare (animal-based measures, resource- and management-based measures, employee resources and training), there would need to be targeted efforts to identify other objective measures, such as comparing severity scores across different studies with the scores from the room-based form, or using employee satisfaction surveys compared with the culture of care form scores. Because the assessments are conducted at least six months apart, measures of reliability have also not been performed. The facilities have rotating animals and studies, and therefore, conducting the assessment within the same room with the same animals over time is difficult to arrange logistically. At any moment, there may be studies with different objectives that could impact animal-based measures of welfare, making test–retest reliability assessment difficult. The benchmarking assessment is meant to capture an average score based on changing studies six months apart to provide a baseline for comparison for future assessments. During the benchmarking assessment, facilities are instructed to use the same tool users to ensure consistency and reduce observer bias; however, overtime, employee changes can impact the ability to assess intra-observer reliability. Future work with the species welfare assessment tools will aim to formally assess validity and reliability. Validity and reliability measures may help pinpoint the most valuable items for rabbit welfare and personnel wellbeing, simplify the tool to make it more efficient, and/or identify companion tools for individual animal, severity, and cumulative use assessments to provide a comprehensive package for facilities to use for improved research rabbit welfare.

## 5. Conclusions

In conclusion, a rabbit welfare assessment tool was developed and implemented within a global contract research organization and commercial breeder. The tool was developed as part of a rabbit welfare working group that developed recommendations on best practices for working with research rabbits [36]. The tool successfully evaluated strengths and weaknesses across facilities and business purposes (i.e., breeding, discovery research, safety testing) and identified global gaps in rabbit welfare and management. The tool investigates room-, site-, and personnel-level welfare indicators related to physical, behavioral, environmental, training, procedural, and culture of care factors. The rabbit welfare assessment tool will aid the organization in improving rabbit welfare company-wide and create a culture of continuous animal welfare improvement.

## Figures and Tables

**Table 1:** Description of each category included in the rabbit welfare assessment tool, the subcategories within each category, and the maximum score for each subcategory.

Category	Subcategories	Maximum Score
Physical: assess the clinical health of the rabbits and factors that may influence health	General condition	46
	Nutrition	26
	Pain assessment and mitigation	18
	Records	24
Behavioral: assess normal and abnormal rabbit behavior	Behavioral assessments	24
	Normal behavior	22
	Social behavior	42
	Feeding behavior	8
	Parental behavior	12
	Bonus	+5
Environmental: assess the quality of the space and resources and ability to express rabbit-typical behaviors and postures	Housing	68
	Resources	86
	Exercise opportunities	16
	Bonus	+5
Training: assess how rabbits are prepared for the research environment to reduce stress and promote cooperation	Acclimation	6
	Habituation and training	84
	Human interactions	56
	Animal cooperation	24
Procedural: assess the procedures and techniques used at the facility to ensure they are in line with the recent literature on optimal practices for rabbits	Restraint	26
	Procedures	60
	Recovery	6
	Ambience	12
	Scheduling	6
	Bonus	+5
Culture of care: assess the training and resources provided to the employees working with rabbits to allow them to feel job satisfaction and confidence in their work with rabbits and to promote compassion satisfaction and resilience	Initial training	56
	Continuing education	12
	Compassion fatigue and resiliency building programs/activities	42
	Involvement/opportunity	16
	Choice and control in work schedule	32
	Recognition	16
	Voice concerns	24
	Competencies	14
	Bonus	+24

**Table 2:** Full list of Rabbit Welfare Assessment Tool descriptors with respective welfare weights, and which form it was included on (room, site, or Culture of Care). The table is divided by category: (a) physical, (b) behavioral, (c) environment, (d) training, (e) procedural, and (f) culture of care.

Descriptor	Subcategory	Form	Weight
(a): Physical category			
Animals are displaying normal posture (i.e., back is not arched head is above shoulders, front paws are untucked)	General condition	Room	4
Animals have a clean and largely intact hair coat	General condition	Room	2
Animals appear to be well hydrated	General condition	Room	3
Animals are bright, alert, and responsive (i.e., reacting normally to stimuli)	General condition	Room	4
Animals are free of obvious wounds or health conditions (e.g., no signs of gastrointestinal, skin, respiratory conditions, normal gait, no swelling)	General condition	Room	4
Animals have appropriate body condition (i.e., muscular development and fat deposit) for sex and age	General condition	Room	3
Animals’ teeth are an appropriate length and condition	General condition	Room	3
Procedures are in place to empty and clean feeders (especially for animals fed ad libitum)	Nutrition	Site	3
Feeding programs are evaluated regularly	Nutrition	Site	3
There are established procedures in place for animals with reduced appetite or body weight (e.g., supplementation)	Nutrition	Site	3
Animals readily consume base diet (e.g., lack of excessive base diet present in enclosure, food consumption monitored for animals fed ad libitum)	Nutrition	Room	4
Care personnel are trained in recognizing species-specific signs of pain	Pain	Site	3
Animals receive individualized dosages of pain medications (i.e., per g or kg)	Pain	Site	3
The facility has at least one specific policy or SOP concerning species-specific pain management practices based on current veterinary practices	Pain	Site	3
Body weight histories are appropriate for sex and age	Records	Room	2
Animals receive prompt veterinary attention when a problem is reported and follow-up until cases are closed	Records	Room	5
Rearing history, nursery foster mother, family group are readily available	Records	Room	2
Complete medical records are readily available for animals in training colonies or unassigned to a study	Records	Room	3
(b): Behavioral category			
Behavioral assessments are conducted regularly for all animals	Behavioral assessments	Site	4
Personnel are trained to identify normal and abnormal behaviors for species	Behavioral assessments	Site	4
There is a team or individual that specializes in species-specific behavior	Behavioral assessments	Site	4
If abnormal behavior is observed, procedures are in place to mitigate or reduce occurrence	Normal behavior	Site	3
Animals are displaying normal behavior	Normal behavior	Room	4
There is no evidence of abnormal behaviors (i.e., signs of hair plucking, self-mutilation, wounds or lesions indicative of other forms of abnormal behavior or stereotypies)	Normal behavior	Room	4
Compatibility of social groupings is assessed regularly (i.e., social groups are checked for signs of positive social behaviors or signs of fighting)	Social behavior	Site	3
Quality of life is assessed for low-ranking animals (e.g., low ranking animal has access to resources and food items, sits in close proximity to other animals)	Social behavior	Site	3
There is an established procedure if social partners are incompatible	Social behavior	Site	3
Animals are housed with at least one other compatible social partner	Social behavior	Room	3
There are no signs of significant fighting such as open wounds, large numbers of scabs, etc.	Social behavior	Room	3
Animals engage in positive social activities, such as sharing, playing or interacting with conspecifics	Social behavior	Room	3
All animals appear to have access to provided resources (i.e., no evidence of animal being bullied or not being able to access food)	Social behavior	Room	3
Food and food resources (treats) are presented in a way that encourages natural behaviors (i.e., foraging, variety, gnawing)	Feeding behavior	Room	4
Animals have the opportunity to express parental behavior	Parental behavior	Room	3
Animals are reared within a natural timespan according to best practice for species (i.e., healthy kits weaned at recommended age of ~35 days old)	Parental behavior	Room	3
The facility has a monitoring system permitting indirect observations (limiting observer effect on animal behavior)	Bonus	Site	5
(c): Environmental category			
When animals are singly housed, extra resources or positive human interaction are required	Housing	Site	4
Animals can walk, jump, run, and stretch (vertically and laterally)	Housing	Room	5
Enclosure allows for natural postural changes for the size/age of animal	Housing	Room	5
There are solid surfaces for animals to rest on	Housing	Room	4
The enclosures have visual barriers and privacy areas for animals to get away from conspecifics and humans	Housing	Room	5
There are multiple feeding, drinking and resting areas in enclosures to maximize access to resources	Housing	Room	3
Substrate is provided (i.e., at least a light layer on bottom of enclosure)	Housing	Room	2
When separated, animals still have visual, auditory, olfactory, and/or tactile contact with conspecifics	Housing	Room	3
The room that animals are housed in is not excessively noisy (e.g., away from crowds, construction, vocalizations from other species, or noise mitigation procedures or equipment are in place)	Housing	Room	3
There is an established rotating resource schedule for the animals (i.e., to ensure variety and stability in resource provision and to prevent animal boredom)	Resources	Site	3
Resource provision is documented	Resources	Site	3
Resource provision is regularly evaluated	Resources	Site	3
There are behavioral management guidelines for species	Resources	Site	4
Behavioral management programs are regularly evaluated	Resources	Site	4
Animals engage with in-cage resources (via direct observation by animal personnel or indirect monitoring)	Resources	Room	5
Thermoneutral surfaces (i.e., surfaces that do not alter animal body temperature) are present to promote comfort	Resources	Room	4
Animals are provided opportunities to forage	Resources	Room	4
Animals are provided opportunities to chew	Resources	Room	5
Animals are provided a grass-type hay (e.g., timothy)	Resources	Room	4
There are sufficient resources for all animals in enclosure	Resources	Room	4
Enclosure space exceeds minimum recommendations (per local specifications)	Exercise opportunities	Room	4
Animals have regular access to additional space outside of their home enclosure for exercise (i.e., an area with additional resources and opportunities for natural behaviors)	Exercise opportunities	Room	4
Animals have access to substrate to dig in (i.e., deep substrate of approximately 8–12 inches within enclosure or offered in exercise pen with regular access) Can be provided with a dig box if the enclosure or exercise pen does not accommodate this.	Bonus	Room	5
(d): Training category			
Site has a pre-determined environmental acclimation period for species (i.e., animals are given time to adjust to environment after arrival, and this time is free from study activities. Habituation activities are allowed during this time)	Acclimation	Site	3
Site has a program (or multiple programs) specifying habituation, desensitization, and/or counter conditioning of animals for procedures (e.g., handling, oral gavage, dosing)	Habituation and training	Site	5
Site has an established program focused on positive reinforcement training techniques for personnel working with animals	Habituation and training	Site	4
Habituation and training programs are regularly evaluated	Habituation and training	Site	3
Staff performing habituation and training activities are properly trained (i.e., training includes purpose, how to perform correctly, and timing of rewards)	Habituation and training	Site	5
Animals are habituated to any/all restraint devices or methods (e.g., chair, sling, jacket, tether, collars, manual)	Habituation and training	Site	4
Habituation is performed in a controlled, quiet environment	Habituation and training	Site	3
Habituation is not paired with other activities (e.g., body weights, blood draws)	Habituation and training	Site	3
Staff document animal behaviour and progress during habituation and training	Habituation and training	Site	3
Habituation and training is maintained throughout the animals’ time at the facility (i.e., between studies or for long term placements)	Habituation and training	Site	3
There are procedures in place for animals not habituating well	Habituation and training	Site	3
At completion of habituation, animals tolerate the procedure	Habituation and training	Room	3
Animals are trained for behaviors that facilitate husbandry or veterinary procedures (e.g., shifting, human interaction) and/or for cognitive stimulation using positive reinforcement	Habituation and training	Room	3
Human interaction program is available species that defines how to build trust and maintain positive interactions with animals	Human interactions	Site	3
Animals are introduced to positive human interactions (e.g., desensitization, gentle touch, offering treats at front of cage) on the day of arrival	Human interactions	Site	3
Animals receive positive human interactions throughout their time at the facility	Human interactions	Site	3
Personnel document animals not responding well to human interactions	Human interactions	Site	2
Same staff are specifically assigned to the same animal rooms to ensure familiarity of animals to people and people to animal needs and to build trust	Human interactions	Site	4
Animals come to the front of the enclosure to interact with humans (i.e., willing to accept treats or voluntarily approach)	Human interactions	Room	3
Animals are calm in the presence of humans (i.e., not showing stress or fear behaviors)	Human interactions	Room	5
Animals are handled with least aversive methods (no scruffing)	Human interactions	Room	5
Animals are comfortable and compliant with handling (do not show signs of fear or distress, e.g., no vocalizations, no struggling to get away, no attempts to bite, no urination/defecation)	Animal cooperation	Room	4
Animals are calm when undergoing procedures (no vocalizations, no struggling to get away, no attempts to bite, no urination/defecation)	Animal cooperation	Room	4
Animals are calm when placed in a restraint device (no vocalizations, no struggling to get away, no attempts to bite, no urination/defecation)	Animal cooperation	Room	4
(e): Procedural category			
Manual restraint provides full support to the animals’ head and body weight	Restraint	Site	3
Restraint devices/procedures are comfortable (i.e., the animal has support in a natural position, soft surfaces, thermoneutral surfaces (e.g., plastic or wood or vet bed))	Restraint	Site	4
For prolonged restraint, there are procedures in place to keep animals comfortable and/or occupied	Restraint	Site	3
If prolonged restraint will occur, there is sufficient staffing to continuously monitor the animals	Restraint	Site	3
Staff are trained to respond appropriately to adverse situations for species (e.g., CPR, hemostasis)	Procedures	Site	3
For repeated blood collections, catheters are used whenever possible	Procedures	Site	4
Training to procedures with positive reinforcement is maintained throughout the animals’ time at the facility (i.e., between studies or for long term placements)	Procedures	Site	3
When removing food from animals for study activities, social housing is maintained	Procedures	Site	3
When separation is needed for an activity/veterinary care, animals are separated right before the procedure and returned to social housing as soon as possible afterwards	Procedures	Site	3
Procedures are scheduled together as much as possible to minimize disturbance of animals	Procedures	Site	2
There is a policy/policies specifying the maximum duration of time, number of uses, or breeding cycles that the animals are maintained (cumulative use/humane endpoints)	Procedures	Site	3
Facility has an active IACUC/AOB approved program for the adoption and rehoming of animals, when possible	Procedures	Site	5
Animals are provided a reward following the procedure	Procedures	Room	4
Following procedures, animals are monitored regularly to ensure that animals are comfortable (e.g., posture, behavior, injection or blood collection site)	Recovery	Site	3
During procedures, ambient noise levels are low (e.g., human voices, radio, background noise)	Ambience	Room	3
There is sufficient space in the procedure areas for animals and humans to move easily without risk of injury	Ambience	Room	3
There are sufficient personnel scheduled to conduct study activities to avoid rushing	Scheduling	Site	3
Animals are prepared for adoption or rehoming using habituation, desensitization, and positive reinforcement training	Bonus	Site	5
(f): Culture of care category			
Personnel are comfortable working with the species they are assigned to	Initial training	CoC	4
Training includes relevant species-specific behavioral training	Initial training	CoC	5
Personnel are trained to respond to species-specific inappropriate or abnormal behavior	Initial training	CoC	5
Employees are allotted enough time to complete skills training	Initial training	CoC	4
If employees do not feel comfortable after initial training, employees can have additional training or other options to accommodate concerns	Initial training	CoC	3
Training is conducted in an environment conducive to learning (e.g., not rushed, competitive with other trainees, chaotic)	Initial training	CoC	3
Employees are evaluated on competency (animal welfare, animal care, animal behaviour, procedures) following training(s) and if competency is poor, aspects of the training are repeated	Initial training	CoC	4
Personnel can complete continuing education during normal working hours	Continuing education	CoC	3
Species-specific resources and continuing education materials are readily accessible for employees	Continuing education	CoC	3
Employees feel that they have a good work life balance	CF/RBA	CoC	5
Employees can step away from an activity when feeling emotionally overwhelmed and there is an established line of communication when this occurs	CF/RBA	CoC	3
Site has an active compassion fatigue/resiliency building program	CF/RBA	CoC	5
Personnel can identify their Resiliency Building Advocates	CF/RBA	CoC	3
Site-specific events occur to honor the animals that employees work with daily	CF/RBA	CoC	2
Employees have access to resources on compassion fatigue and resiliency building strategies	CF/RBA	CoC	3
Employees actively participate in resource evaluation and improvement (e.g., new manipulanda, caging or food stuff)	Involvement/opportunity	CoC	3
Employees are actively involved in method development and procedure refinement	Involvement/opportunity	CoC	3
Technical staff are encouraged and given time to attend/participate at pre-study meetings and can provide input on the study plan	Involvement/opportunity	CoC	2
Employees have sufficient time to complete procedures and do not feel rushed	Choice and control	CoC	5
Employees have time for positive interactions with the animals	Choice and control	CoC	5
Employees can choose to specialize with a preferred species	Choice and control	CoC	3
Employees can choose to opt-out of performing activities that they are uncomfortable doing without repercussions (e.g., euthanizing familiar animals)	Choice and control	CoC	3
Site-specific award program is present to recognize employee excellence when working with animals	Recognition	CoC	4
Employees feel valued for their work	Recognition	CoC	4
Employees are comfortable reporting animal welfare concerns without fear of reprisal	Voice concerns	CoC	4
Technical personnel have an equal or valued role when concerns are raised	Voice concerns	CoC	4
Employees feel their concerns are addressed	Voice concerns	CoC	4
The facility staff participates in meetings with other sites to discuss challenges and refinements	Competencies	CoC	3
Techniques are constantly being evaluated, in line with current and emerging laboratory animal science practices and literature	Competencies	CoC	2
Technical staff have a platform to ask questions, comment, or share information regarding procedures	Competencies	CoC	1
Technical staff can readily access SOP/BOP during their day-to-day work	Competencies	CoC	1
Site offers access to physical activities at times in which all staff can attend (e.g., access to fitness facilities/paid gym membership, on-site yoga classes)	Bonus	CoC	7
Supervisors have attended the Frontline Leaders Workshop on creating an emotionally engaged culture through empathetic listening and the CR C.A.R.E.S. program	Bonus	CoC	7
Site has an active adoption or rehoming programs for relevant species	Bonus	CoC	10

CF/RBA: Compassion fatigue and resiliency building programs/activities.

**Table 3:** The rabbit welfare assessment score (presented as percentage based on score achieved/total maximum score) for each facility and the average global scores for each category and subcategory. Each facility is denoted by a different letter.

Facility	A			B		C		D		E		F	
Category	Q1			Q1	Q3	Q1	Q3	Q1	Q3	Q1	Q3	Q1	Q3
Physical	100			98	96	97	99	97	97	100	100	98	98
Conditions	100			100	100	99	100	99	100	100	100	100	100
Nutrition	100			100	93	92	100	100	93	100	100	86	86
Pain	100			67	50	83	83	67	67	200	100	100	100
Records	100			100	100	100	100	100	100	100	100	100	100
Behavioral	96			96	83	90	87	90	93	88	89	87	91
Assessments	83			100	33	83	67	67	67	67	67	83	83
Normal Behavior	100			100	100	100	100	100	100	100	100	100	100
Social	100			90	83	79	74	83	90	82	80	70	80
Feeding	100			100	67	100	100	100	100	100	100	83	83
Parental	NA			100	100	100	100	100	100	NA	NA	NA	NA
Bonus	0			0	0	0	5	0	0	0	0	5	5
Environmental	98			90	95	80	88	79	78	83	82	66	64
Housing	100			84	93	79	96	82	72	78	80	61	54
Resources	100			86	89	95	96	90	98	94	93	79	84
Exercise	50			100	100	25	25	25	25	50	50	33	25
Bonus +	5			15	10	0	0	0	0	0	0	0	0
Bonus −	0			0	0	0	0	0	0	0	0	0	0
Training	81			65	77	75	79	71	76	50	72	91	86
Acclimation	100			100	100	100	100	100	100	100	100	100	100
Habituation/training	83			42	61	60	64	54	55	20	63	79	71
Human interaction	68			70	80	70	76	75	81	63	66	96	92
Animal cooperation	100			89	94	100	100	89	100	83	94	100	100
Procedural	87			77	83	74	82	75	74	83	83	73	75
Restraint	100			100	88	100	100	62	62	100	100	88	77
Procedures	77			47	63	73	79	68	74	68	67	57	76
Recovery	100			100	100	100	100	100	100	100	100	50	50
Ambience	100			100	100	63	69	88	83	100	100	100	75
Scheduling	100			100	50	50	50	50	50	100	100	0	0
Bonus	0			5	5	0	5	0	0	0	0	5	5
**Facility**	**G**		**H**		**I**	**J**	**K**	**L**		**M**		**Average**	
Category	Q1	Q3	Q1	Q3	Q1	Q3	Q1	Q1	Q3	Q1	Q3	Q1	Q3
Physical	94	100	99	100	93	100	93	98	100	95	95	97	98
Conditions	96	100	100	100	99	100	100	96	100	100	100	99	100
Nutrition	100	100	100	100	69	100	100	100	100	82	82	94	95
Pain	100	100	83	100	100	100	100	100	100	100	100	92	89
Records	86	100	98	100	93	100	75	100	100	100	100	95	100
Behavioral	84	72	81	89	91	83	78	76	92	81	97	87	88
Assessments	100	100	50	83	100	50	67	100	100	67	100	81	72
Normal Behavior	93	100	100	94	94	100	100	100	100	100	100	99	99
Social	73	63	82	83	80	86	80	71	79	71	93	80	82
Feeding	67	67	38	83	100	100	0	0	100	100	100	74	87
Parental	100	100	94	100	NA	NA	NA	50	100	100	100	98	100
Bonus	5	0	0	0	0	0	0	0	0	0	0		
Environmental	70	75	73	55	73	64	69	68	93	48	65	75	77
Housing	74	80	71	62	91	51	74	63	100	50	81	76	77
Resources	78	85	78	49	67	80	74	79	94	51	60	82	84
Exercise	25	25	63	50	25	25	25	25	25	25	25	40	41
Bonus +	0	0	0	0	0	0	0	0	5	0	0		
Bonus −	0	0	0	0	0	0	0	0	0	0	0		
Training	86	94	79	91	71	68	77	47	52	76	61	73	76
Acclimation	NA	NA	100	100	50	100	100	100	100	NA	NA	96	100
Habituation/training	84	84	76	84	48	74	69	33	40	67	67	60	65
Human interaction	79	79	66	91	83	63	69	41	46	77	54	73	76
Animal cooperation	100	83	100	100	89	50	100	100	100	100	50	96	93
Procedural	73	60	79	79	72	94	79	88	90	57	77	76	81
Restraint	NA	NA	100	73	85	100	100	85	85	NA	NA	88	85
Procedures	74	74	63	74	54	80	57	82	80	58	93	64	75
Recovery	NA	NA	100	50	100	100	100	100	100	NA	NA	96	78
Ambience	58	100	88	100	100	100	100	75	100	0	75	85	88
Scheduling	100	100	50	0	50	100	50	100	100	0	0	63	56
Bonus	5	0	5	5	0	5	5	5	5	0	0		
**Facility**	**B**			**C**		**D**		**E**		**F**		**G**	
Category	Q1		Q3	Q1	Q3	Q1	Q3	Q1	Q3	Q1	Q3	Q1	Q3
Culture of care	77		87	66	82	82	69	87	78	81	75	70	62
Initial training	91		97	69	93	83	73	90	92	90	77	86	82
Continuing education	75		100	63	92	69	70	92	92	83	75	100	100
CF/RBA	31		58	60	67	76	68	70	39	73	83	48	45
Involvement/opportunity	63		81	56	67	78	63	100	100	69	58	63	63
Choice and control	78		81	51	67	74	52	89	82	50	45	56	19
Recognition	75		83	75	92	81	75	67	42	92	83	63	50
Voice concerns	89		100	64	86	83	79	94	94	8	67	58	67
Competencies	93		67	77	86	82	68	57	45	98	88	79	100
Bonus	24		24	24	24	20	24	24	24	20	20	10	0
**Facility**	**H**			**I**	**J**		**K**	**L**		**M**		**Average**	
Category	Q1		Q3	Q1	Q1	Q3	Q1	Q1	Q3	Q1	Q3	Q1	Q3
Culture of care	75		73	80	72	71	79	97	95	95	87	75	74
Initial training	89		89	88	96	82	98	100	100	98	100	87	84
Continuing education	65		50	75	83	58	75	100	100	50	100	74	78
CF/RBA	69		75	56	52	62	48	95	95	90	83	63	67
Involvement/opportunity	78		56	77	48	65	79	100	100	100	63	74	68
Choice and control	52		46	81	51	45	61	84	75	79	56	65	57
Recognition	85		69	83	58	67	75	75	75	100	75	78	74
Voice concerns	63		79	88	61	67	94	83	83	100	83	78	81
Competencies	67		55	52	71	88	64	86	86	95	79	76	73
Bonus	24		24	24	24	24	24	10	10	24	10		

CF/RBA: Compassion fatigue and resiliency building programs/activities.

**Table 4:** Recommendations for improvement for each category based on assessment results.

Category	Recommendations
Physical	-Develop training on signs of pain in rabbits for personnel working with rabbits. -Review policies on pain management to ensure in line with current veterinary recommendations for rabbits.
Behavioral	-Implement regular, proactive behavioral assessments for rabbits. -Introduce more social housing for rabbits, or look for opportunities for protected social contact for animals that cannot be socially housed.
Environmental	-Provide more visual barriers in housing for rabbits to get away from humans and conspecifics. -Provide thermoneutral surfaces for rabbits to rest on. -Provide substrate within home enclosure. -If housing does not provide opportunities for natural behaviors and postures, provide exercise opportunities outside of home enclosure. -Develop formalized behavioral management program for rabbits. -Implement a rotating resource schedule. -Provide foraging and chewing opportunities and monitor animals to ensure resources are being used as intended.
Training	-Develop programs for personnel on how to habituate and train rabbits, and how to build positive relationships. -Formalize habituation and training programs, implementing more habituation to procedures and positive reinforcement training to encourage cooperation. -Document animals not responding well and needing additional attention.
Procedural	-Provide rewards after procedures. -Develop policy on cumulative use and humane endpoints. -Use catheters for procedures that require repeat blood collections. -Evaluate scheduling practices to reduce personnel feeling rushed.
Culture of care	-Implement formal program on compassion fatigue and resiliency building and/or raise awareness of existing resources. -Implement and communicate protocol for allowing personnel to opt out of practices when uncomfortable or overwhelmed. -Provide personnel a quiet place.

## Data Availability

The original contributions presented in this study are included in the article/Supplementary Materials. Further inquiries can be directed to the corresponding author.

## Supplementary Materials

The following supporting information can be downloaded at: https://www.mdpi.com/article/10.3390/ani16081229/s1, Table S1: RbtWAT Worksheet–Room level–EN; Table S2: RbtWAT Worksheet–Site level–EN; Table S3: CoC Site Worksheets–English.
