# Supplementary material for: A Welfare Assessment Tool to Harmonize Care and Management for Research Rabbits
Source: Animals (Basel). 2026 Apr 17;16(8):1229. doi: 10.3390/ani16081229 (PMC13114067; doi:10.3390/ani16081229)
Supplement: Supplementary file 1 [file animals-16-01229-s001.zip › S2 RbtWAT Worksheet - Site level - EN.pdf]

**Physical - Nutrition**	**2**	**1**	**0**	**NA**
Procedures are in place to empty and clean feeders (especially for animals fed ad libitum)	yes	for some animals/sometimes	no	
Feeding programs are evaluated regularly	yes	for some animals/sometimes	no	
There are established procedures in place for animals with reduced appetite or body weight (e.g., supplementation)	yes	for some animals/sometimes	no	
**Physical - Pain assessment and mitigation**	**2**	**1**	**0**	
Care personnel are trained in recognizing species-specific signs of pain	yes	some personnel	no	
Animals receive individualized dosages of pain medications (i.e., per g or kg)	yes	for some animals/sometimes	no	
The facility has at least one specific policy or SOP concerning species-specific pain management practices based on current veterinary practices	yes	for some animals/sometimes	no	
**Behavioral - Behavioral assessments**				
Behavioral assessments are conducted regularly for all animals	yes	for some animals/sometimes	no	
Personnel are trained to identify normal and abnormal behaviors for species	yes	some personnel	no	
There is a team or individual that specializes in species-specific behavior	yes	no		
**Behavioral - Animal behavior**	**2**	**1**	**0**	
If abnormal behavior is observed, procedures are in place to mitigate or reduce occurrence	yes	for some animals/sometimes	no	
**Behavioral - Social behavior**	**2**	**1**	**0**	**NA**
Compatability of social groupings is assessed regularly	yes	for some animals/sometimes	no	not socially housed
Quality of life is assessed for low ranking animals (e.g., low ranking animal has access to resources and food items, sits in close proximity to other animals)	yes	for some animals/sometimes	no	not socially housed
There is an established procedure if social partners are incompatible	yes	for some animals/sometimes	no	not socially housed
**Behavioral - Bonus**				
The facility has a monitoring system permitting indirect observations (limiting observer effect on animal behavior)	yes	no		
**Environmental - Housing**	**2**	**1**	**0**	**NA**
When animals are singly housed, extra resources or positive human interaction are required	yes	for some animals/sometimes	no	not singly housed
**Environmental - Resources**	**2**	**1**	**0**	
There is an established rotating resource schedule for the animals (i.e., to ensure variety and stability in resource provision and to prevent animal boredom)	yes	for some animals/sometimes	no	
Resource provision is documented	yes	for some animals/sometimes	no	
Resource provision is regularly evaluated	yes	for some animals/sometimes	no	
There are behavioral management guidelines for species	yes	for some animals/sometimes	no	
Behavioral management programs are regularly evaluated	yes	for some animals/sometimes	no	
**Training - Acclimation**	**2**	**1**	**0**	
Site has a pre-determined environmental acclimation period for species (i.e, animals are given time to adjust to environment after arrival and this time is free from study activities. Habituation activites are allowed during this time)	yes	for some animals/sometimes	no	
**Training - Habituation and training**	**2**	**1**	**0**	**NA**

Site has a program (or multiple programs) specifying habituation, desensitization, and/or counter conditioning of animals for procedures (e.g., handling, oral gavage, dosing)	yes	for some animals/sometimes	no	
Site has an established program focused on positive reinforcement training techniques for personnel working with animals	yes	for some animals/sometimes	no	
Habituation and training programs are regularly evaluated	yes	for some animals/sometimes	no	
Staff performing habituation and training activities are properly trained (i.e., training includes purpose, how to perform correctly, and timing of rewards)	yes	some personnel	no	
Animals are habituated to any/all restraint devices or methods (e.g., chair, sling, jacket, tether, collars, manual)	yes	for some animals/sometimes	no	not restrained
Habituation is performed in a controlled, quiet environment	yes	for some animals/sometimes	no	
Habituation is not paired with other activities (e.g., body weights, blood draws)	yes	for some animals/sometimes	no	
Staff document animal behaviour and progress during habituation and training	yes	for some animals/sometimes	no	
Habituation and training is maintained throughout the animals' time at the facility (i.e., between studies or for long term placements)	yes	for some animals/sometimes	no	
There are procedures in place for animals not habituating well	yes	for some animals/sometimes	no	
Positive reinforcement training is used for animals housed long-term and/or for those in more challenging studies	yes	some animals	no	no long term studies
**Training - Human interactions**	**2**	**1**	**0**	
Human interaction program is available for species that defines how to build trust and maintain positive interactions with animals	yes	for some animals/sometimes	no	
Animals are introduced to positive human interactions (e.g., desensitization, gentle touch, offering treats at front of cage) on the day of arrival	yes	for some animals/sometimes	no	
Animals receive positive human interactions throughout their time at the facility	yes	for some animals/sometimes	no	
Personnel document animals not responding well to human interactions	yes	for some animals/sometimes	no	
Same staff are specifically assigned to the same animal rooms to ensure familiarity of animals to people and people to animal needs and to build trust	yes	for some animals/sometimes	no	
**Procedural - Restraint**	**2**	**1**	**0**	**NA**
Manual restraint provides full support to the animals' head and body weight	yes	for some animals/sometimes	no	
Restraint devices/procedures are comfortable (i.e., the animal has support in a natural position, soft surfaces, thermoneutral surfaces (e.g., plastic or wood or vet bed))	yes	for some animals/sometimes	no	
For prolonged restraint, there are procedures in place to keep animals comfortable and/or occupied	yes	for some animals/sometimes	no	no prolonged restraint
If prolonged restraint will occur, there is sufficient staffing to continously monitor the animals	yes	for some animals/sometimes	no	no prolonged restraint
**Procedural - Procedures**	**2**	**1**	**0**	**NA**
Staff are trained to respond appropriately to adverse situations for species (e.g., CPR, hemostasis)	yes	some personnel	no	
For repeated blood collections, catheters are used whenever possible	yes	for some animals/sometimes	no	no repeat blood collection
Training to procedures with positive reinforcement is maintained throughout the animals' time at the facility (i.e., between studies or for long term placements)	yes	for some animals/sometimes	no	
When removing food from animals for study activities, social housing is maintained	yes	for some animals/sometimes	no	no social housing

When separation is needed for an activity/veterinary care, animals are separated right before the procedure and returned to social housing as soon as possible afterwards	yes	for some animals/sometimes	no	no social housing
Procedures are scheduled together as much as possible to minimize disturbance of animals	yes	for some animals/sometimes	no	
There is a policy/policies specifying the maximum duration of time, number of uses, or breeding cycles that the animals are maintained (cumulative use/humane endpoints)	yes	for some animals/sometimes	no	not applicable
Facility has an active IACUC/AOB approved program for the adoption and rehoming of animals, when possible	yes	for some animals/sometimes	no	not possible
**Procedural - Recovery**	**2**	**1**	**0**	
Following procedures, animals are monitored regularly to ensure that animals are comfortable (e.g., posture, behavior, injection or blood collection site)	yes	for some animals/sometimes	no	
**Procedural - Scheduling**	**2**	**1**	**0**	
There are sufficient personnel scheduled to conduct study activities to avoid rushing	yes	for some animals/sometimes	no	
**Bonus**				
Reprogramming of animals occurs routinely prior to adoption or rehoming	yes	no		
