# Supplementary material for: A Welfare Assessment Tool to Harmonize Care and Management for Research Rabbits
Source: Animals (Basel). 2026 Apr 17;16(8):1229. doi: 10.3390/ani16081229 (PMC13114067; doi:10.3390/ani16081229)
Supplement: Supplementary file 1 [file animals-16-01229-s001.zip › S3 CoC Site Worksheets - English.pdf]

**Initial training**	**2**	**1**	**0**	**NA**
Personnel are comfortable working with the species they are assigned to	yes	occasionally	no	
Training includes relevant species-specific behavioral training	yes	for some species	no	
Personnel are trained to respond to species-specific inappropriate or abnormal behavior	yes	for some species	no	
Employees are allotted enough time to complete skills training	yes	occasionally	no	
If employees do not feel comfortable after initial training, employees can have additional training or other options to accommodate concerns	yes	occasionally	no	
Training is conducted in an environment conducive to learning (e.g., not rushed, competitive with other trainees, chaotic)	yes	occasionally	no	
Employees are evaluated on competency (animal welfare, animal care, animal behaviour, procedures) following training(s) and if competency is poor, aspects of the training are repeated	yes	occasionally	no	
**Continuing education**	**2**	**1**	**0**	
Personnel can complete continuing education during normal working hours	yes	occasionally	no	
Species-specific resources and continuing education materials are readily accessible for employees	yes	occasionally	no	
**Compassion fatigue/Resilience Building Program/Activities**	**2**	**1**	**0**	
Employees feel that they have a good work life balance	yes	occasionally	no	
Employees can step away from an activity when feeling emotionally overwhelmed and there is an established line of communication when this occurs	yes	occasionally	no	
Site has an active compassion fatigue/resiliency building program	yes	occasionally	no	
Personnel can identify their Resiliency Building Advocates	yes	occasionally	no	NA
Site-specific events occur to honor the animals that employees work with daily	yes	occasionally	no	
Employees have access to resources on compassion fatigue and resiliency building strategies	yes	occasionally	no	
**Involvement/Opportunity**	**2**	**1**	**0**	
Employees actively participate in resource evaluation and improvement (e.g., new manipulanda, caging or food stuff)	yes	occasionally	no	
Employees are actively involved in method development and procedure refinement	yes	occasionally	no	
Technical staff are encouraged and given time to attend/participate at pre-study meetings and can provide input on the study plan	yes	occasionally	no	NA
**Choice and control in work/schedule**	**2**	**1**	**0**	
Employees have sufficient time to complete procedures and do not feel rushed	yes	occasionally	no	
Employees have time for positive interactions with the animals	yes	occasionally	no	
Employees can choose to specialize with a preferred species	yes	occasionally	no	NA
Employees can choose to opt-out of performing activities that they are uncomfortable doing without repercussions (e.g., euthanizing familiar animals)	yes	occasionally	no	

**Recognition**	**2**	**1**	**0**
Site-specific award program is present to recognize employee excellence when working with animals	yes	occasionally	no
Employees feel valued for their work	yes	occasionally	no
**Voice concerns**	**2**	**1**	**0**
Employees are comfortable reporting animal welfare concerns without fear of reprisal	yes	occasionally	no
Technical personnel have an equal or valued role when concerns are raised	yes	occasionally	no
Employees feel their concerns are addressed	yes	occasionally	no
**Competencies**	**2**	**1**	**0**
The facility staff participates in meetings with other sites to discuss challenges and refinements	yes	occasionally	no
Techniques are constantly being evaluated, in line with current and emerging laboratory animal science practices and literature	yes	occasionally	no
Technical staff have a platform to ask questions, comment, or share information regarding procedures	yes	occasionally	no
Technical staff can readily access SOP/BOP during their day to day work	yes	occasionally	no

**Additional scoring items (in room or building)**		
Site offers access to physical activities at times in which all staff can attend (e.g., access to fitness facilities/paid gym membership, on-site yoga classes)	yes	no
Supervisors have attended the Frontline Leaders Workshop on creating an emotionally engaged culture through empathetic listening and the CR C.A.R.E.S. program	yes	no
Site has an active adoption or rehoming programs for relevant species	yes	no
