# Supplementary material for: A Welfare Assessment Tool to Harmonize Care and Management for Research Rabbits
Source: Animals (Basel). 2026 Apr 17;16(8):1229. doi: 10.3390/ani16081229 (PMC13114067; doi:10.3390/ani16081229)
Supplement: Supplementary file 1 [file animals-16-01229-s001.zip › S1 RbtWAT Worksheet - Room level - EN.pdf]

Physical - General condition	2	1	0	NA
Animals are displaying normal posture (i.e., back is not arched, head is above shoulders, front paws are untucked)	agree: most animals have normal posture	somewhat agree	disagree: most animals have abnormal posture	
Animals have a clean and largely intact hair coat	agree: most animals have clean and full coats	somewhat agree	diagree: most animals have poor coat quality	
Animals appear to be well hydrated	agree: most animals appear well hydrated	somewhat agree	disagree: most animals appear dehydated	
Animals are bright, alert, and responsive (i.e., reacting normally to stimuli)	agree: most animals bright, alert, responsive	somewhat agree	disagree: most animals not bright, alert, responsive	
Animals are free of obvious wounds or health conditions (e.g., no signs of gastrointestinal, skin, respiratory conditions, normal gait, no swelling)	agree: most animals don't have obvious wounds or health conditions	somewhat agree	disagree: most animals have obvious wounds or health conditions	
Animals have appropriate body condition (i.e., muscular development and fat deposit) for sex and age	agree: most animals have appropriate body condition	somewhat agree	disagree: most animals have poor body condition	
Animals' teeth are an appropriate length and condition	agree: most animals have appropriate teeth	somewhat agree	disagree: most animals have poor teeth	
**Physical - Nutrition**	**2**	**1**	**0**	**NA**
Animals readily consume base diet (e.g., lack of excessive base diet present in enclosure, food consumption monitored for animals fed ad libitum)	agree: animals eating well	somewhat agree	disagree: animals not eating well	Fasted animals
**Physical - Records**	**2**	**1**	**0**	**NA**
Body weight histories are appropriate for sex and age	agree: most animals have appriopriate weight histories	somewhat agree	disagree: most animals do not have appropriate body weight histories	
Animals receive prompt veterinary attention when a problem is reported and follow-up until cases are closed	yes	sometimes/some animals	no	
Rearing history, nursery foster mother, family group are readily available	yes	some animals	no	Not applicable
Complete medical records are readily available for animals in training colonies or unassigned to a study	yes	some animals	no	Not applicable
**Behavioral - Animal behavior**	**2**	**1**	**0**	
Animals are displaying normal behavior	agree: mostly observed normal behavior	somewhat agree	disagree: most animals showing abnormal behavior	
There is no evidence of abnormal behaviors (i.e., signs of hair plucking, self mutilation, wounds or lesions indicative of other forms of abnormal behavior or stereotypies)	agree: most animals are free of evidence of abnormal behavior	somewhat agree	disagree: most animals showing evidence of abnormal behavior	
**Behavioral - Social behavior**	**2**	**1**	**0**	**NA**
Animals are housed with at least one other compatible social partner	yes: animals socially housed	some: mix of single and social housing	no: animals single housed	
There are no signs of significant fighting such as open wounds, large numbers of scabs, etc.	agree: little evidence of fighting	somewhat agree	disagree: most animals show evidence of fighting	Not socially housed
Animals engage in positive social activities, such as sharing space and resources, playing, or interacting with conspecifics	agree: mostly positive social activities observed	somewhat agree	disagree: not many positive social activities observed	Not socially housed
All animals appear to have access to provided resources (i.e., no evidence of animal being bullied or not being able to access food)	agree: animals have access to resources	somewhat agree	disagree: conflict over resources observed in most enclosures	Not socially housed
**Behavioral - Feeding behavior**	**2**	**1**	**0**	
Food and food resources (treats) are presented in a way that encourages natural behaviors (i.e., foraging, variety, gnawing)	yes	sometimes/some animals	no	
**Behavioral - Parental behavior**	**2**	**1**	**0**	**NA**
Animals have the opportunity to express natural parental behavior	yes	some	no	Not applicable
Animals are reared within a natural timespan according to best practice for species	yes	some	no	Not applicable
**Environmental - Housing**	**2**	**1**	**0**	
Animals can walk, jump, run, and stretch (vertically and laterally)	yes	somewhat	no	
Enclosure allows for natural postural changes for the size/age of animal	yes	somewhat	no	
There are solid surfaces for animals to rest on	yes	somewhat	no	
The enclosures have visual barriers and privacy areas for animals to get away from from conspecifics and humans	yes	some	no	
There are multiple feeding, drinking, and resting areas in enclosures to maximize access to resources	yes	some	no	
Substrate is provided	yes	occasionally	no	
When separated, animals still have visual, auditory, olfactory, and/or tactile contact with conspecifics	yes	some	no	

The room that animals are housed in is not excessively noisy (e.g., away from crowds, construction, vocalizations from other species, or noise mitigation procedures or equipment are in place)	agree: room is quiet	somewhat agree	disagree: room is noisy	
**Environmental - Resources**	**2**	**1**	**0**	
Animals engage with in-cage resources (via direct observation by animal personnel or indirect monitoring)	agree: observed engagement	somewhat agree	disagree: no resource engagement observed	
Thermoneutral surfaces (i.e., surfaces that do not alter animal body temperature) are present to promote comfort	yes	some	no	
Animals are provided opportunities to forage	yes	somewhat/occasionally	no	
Animals are provided opportunities to chew	yes	somewhat/occasionally	no	
There are sufficient resources for all animals in enclosure	yes	somewhat/occasionally	no	Not socially housed
**Environmental - Exercise opportunities**	**2**	**1**	**0**	
Enclosure space exceeds minimum recommendations (per local specifications)	exceeds requirements	meets requirements	below requirements	
Animals have regular access to additional space outside of their home enclosure for exercise (i.e., an area with additional resources and opportunities for natural behaviors; let out of cages during husbandry)	yes, once a week or more	yes, less than once a week	no	
**Environmental Bonus**				
Animals have access to substrate to dig in	yes	no		
**Training - Habituation and training**	**2**	**1**	**0**	
At completion of habituation, animals tolerate the procedure	yes, most or all animals	some animals tolerate	no, most or all animals do not	
Animals are trained for behaviors that facilitate husbandry or veterinary procedures (e.g., shifting, checking waterers, human interaction) and/or for cognitive stimulation using positive reinforcement	yes	some animals	no	
**Training - Human interaction**	**2**	**1**	**0**	
Animals come to the front of the enclosure to interact with humans (i.e., willing to accept treats or voluntarily approach)	yes, most or all animals	some animals	no, most or all animals do not	
Animals are calm in the presence of humans (i.e., not showing stress or fear behaviors)	yes, most or all animals	some animals	no, most or all animals are not	
Animals are handled with least aversive methods (no scruffing)	yes, most or all animals	some animals	no, most or all animals are not	
**Training - Animal cooperation**	**2**	**1**	**0**	**NA**
Animals are comfortable and compliant with handling (do not show signs of fear or distress; e.g., no vocalizations, no struggling to get away, no attempts to bite, no urination/defecation)	yes, most or all animals	some animals	no, most or all animals do not	
Animals are calm when undergoing procedures (no vocalizations, no struggling to get away, no attempts to bite, no urination/defecation)	yes, most or all animals	some animals	no, most or all animals do not	
Animals are calm when placed in a restraint device (no vocalizations, no struggling to get away, no attempts to bite, no urination/defecation)	yes, most or all animals	some animals	no, most or all animals do not	restraint devices not used
**Procedural - Procedures**	**2**	**1**	**0**	
Animals are provided a reward following the procedure	yes, most or all animals	some animals	no, most or all animals do not	
**Procedural - Ambience**	**2**	**1**	**0**	
During procedures, ambient noise levels are low (e.g., human voices, radio, background noise)	yes, noise levels are low	somewhat low	no, it is loud	
There is sufficient space in the procedure areas for animals and humans to move easily without risk of injury	yes, sufficient space	somewhat	no, not enough space	
